# Use of gel caps to aid endoscopic insertion of nasogastric feeding tubes: a comparative audit

**DOI:** 10.1186/1758-3284-3-24

**Published:** 2011-05-07

**Authors:** Tahwinder Upile, Paul Stimpson, Miles Christie, Jaspal Mahil, Hitesh Tailor, Waseem K Jerjes

**Affiliations:** 1 Department of Head and Neck Surgery, University College London Hospital, London, UK; 2 Department of Head and Neck Surgery, Chase Farm & Barnet Hospitals, London, UK; 3 Department of Medicine, University College London Medical School, London, UK; 4 Department of Surgery, University College London Medical School, London, UK

## Abstract

**Introduction:**

Nutrition is crucial to successful outcomes in peri-operative head and neck cancer patients. Nasogastric feeding tubes are an accepted and safe method of providing enteral nutrition in the short-term. Many methods have been advocated for successfully inserting and securing nasogastric tubes and each practitioner will have his or her preferred technique.

**Objectives:**

To confirm the effectiveness of using gel caps combined with the flexible nasendoscope for the insertion of nasogastric feeding tubes in head and neck cancer patients following failure of traditional methods.

**Participants:**

Thirty-five consecutive patients requiring nasogastric feeding tubes were included in this comparative audit. All had failed traditional insertion methods after 2 attempts and were therefore eligible for inclusion. Patients were randomised to undergo attempted insertion with the flexible nasendoscope with or without the use of a gel cap (both methods have been previously described).

**Audit Outcome:**

Primary outcome measures showed no significant difference between the two techniques.

**Discussion:**

We found the methodology to be of no greater benefit to our patients when compared to our alternative current practice for failed blind nasogastric tube insertion. We retain this methodology in our armamentarium for difficult circumstances but have continued with our standard practice for most patients needing nasogastric tube placement.

## Introduction

Nutrition is crucial to successful outcomes in peri-operative head and neck cancer patients. Nasogastric (NG) feeding tubes are an accepted and safe method of providing enteral nutrition in the short-term [1-3]. Many methods have been suggested for successfully inserting and securing nasogastric tubes and each practitioner will have his or her preferred technique. Traditionally, NG feeding tubes have been inserted in a blind fashion and their position confirmed using various methods including pH analysis and/or radiological imaging preferably plain chest X-ray including the upper abdomen [3-7]. Placing nasogastric feeding tubes in patients with head & neck carcinoma can be challenging. These patients may have alterations in the local anatomy of their upper aero-digestive tract due either to the disease process or its treatment (including ablative & reconstructive therapy). This has been known to cause difficulty with 'blind' nasogastric tube placement, often leading to several failed attempts occasionally causing patient discomfort and pain.

Kelly and Lee [2] described a technique whereby a flexible nasendoscope was used to assist in inserting nasogastric tubes under direct vision, thereby negating the requirement for positional checks. This method has been widely adopted for difficult tube insertion and has become the method of choice in the majority of head and neck units where traditional techniques are often unsuccessful due to deranged anatomy and swallowing difficulties. Unfortunately this technique, although an improvement over 'blind' placement, does not allow easy control or manipulation of the direction of movement of the naogastric tube. This maybe overcome by using a variant of the 'gel cap' technique first described by Srouji et al. [2]. This novel method for inserting problematic nasogastric tubes using Blom Singer 16 Fr gel caps, which are designed for the insertion of the post laryngectomy voice restoration system, allows gentle manipulation of both the nasogastric tube and endoscope under vision. Using this technique for some of the patients may lead to successful insertion in certain circumstances.

We decided to assess the effectiveness of the use of these gel caps in conjunction with the flexible nasendoscope versus the use of a flexible nasendoscope alone (which was our standard practice) in inserting nasogastric tubes for patients with head and neck carcinoma. The audit consisted of a randomized protocol to validate the methodology.

## Participants

Both methods of nasogastric tube placement were used in our department. This protocol was carried out as part of the baseline audit spiral. Fully informed consent was obtained from all participants.

Inpatients requiring nasogastric feeding tubes were identified following assessment by the multidisciplinary head and neck team. Thirty-five consecutive patients with head and neck carcinoma were included. The comparative audit took place at the UCLH Head and Neck Unit, London. All patients had failed traditional insertion methods after 2 attempts and were therefore eligible for inclusion.

Nasogastric tube insertion was attempted using an established blind technique whereby a fine bore nasogastric feeding tube is passed into the nose and the patient asked to swallow a small volume of water. The tube is simultaneously advanced into the oesophagus and the position confirmed using pH testing of aspirate and/or chest X-ray.

**Figure 1:**
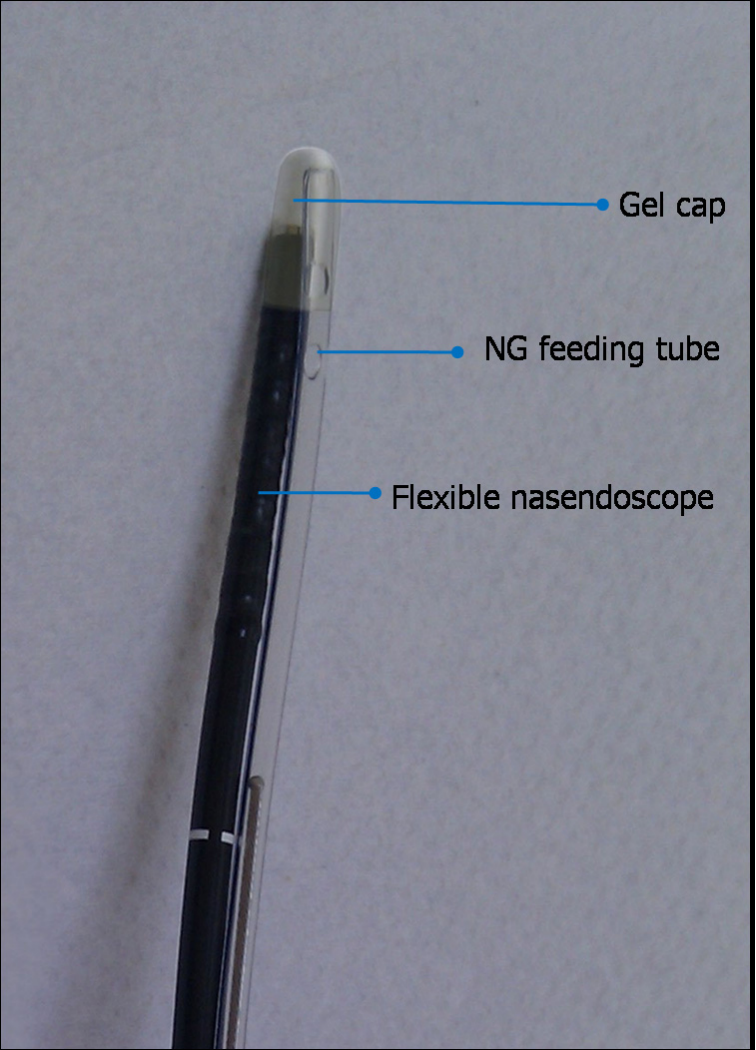
**Feeding tube with the aid of the flexible nasendoscope and gel cap combined**.

**Figure 2:**
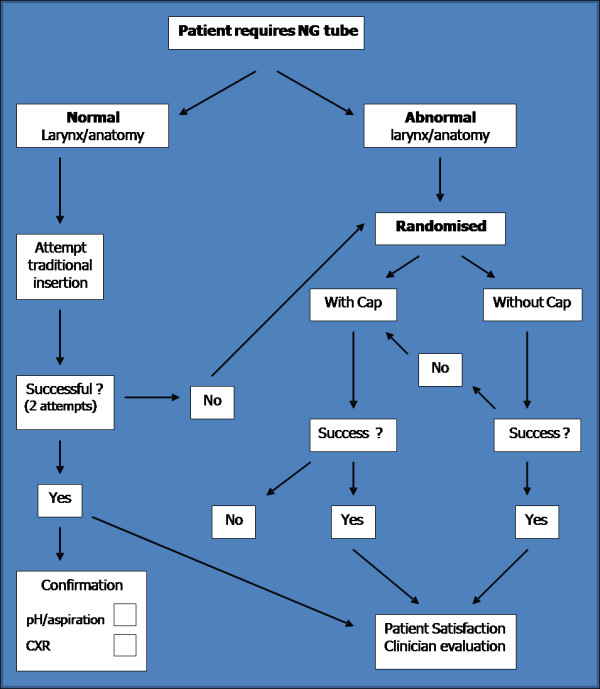
**Flow diagram showing patient recruitment and randomization in the two groups**.

Following 2 failed attempts, patients were then randomised to undergo insertion of feeding tube with the aid of the flexible nasendoscope alone (Group A) or with the flexible nasendoscope and gel cap combined (Group B), (Figure 1). Patients were randomized using a computerized random number generator (0-100) where even numbers were allocated to Group A, and odd numbers to Group B. The randomization was implemented by the clinician performing the procedure immediately before insertion (Figure 2).

All patients received 3 metered doses of co-phenylcaine (lignocaine 5% with phenylephrine) nasal spray with a further 2 doses after an interval of 5 minutes to the side of the nose identified as clearer by the clinician performing the procedure. Water based lubricants were used in both sample groups.

Patients in Group A underwent insertion of the flexible nasendoscope and when an adequate view was achieved, the nasogastric tube was then passed over the endoscope until the tip of the tube was visible in the nasopharynx. The tube could then be advanced and observed to pass into the oesophagus under direct vision. Patients in Group B underwent simultaneous insertion of flexible nasendoscope and feeding tube. The tip of the feeding tube and the tip of the flexible nasendoscope were inserted together into a gel cap (Figure 1). The feeding tube and nasendoscope were then passed together through the nose and directly into the pyriform fossa and advanced into the oesophagus. Both were then left in place for 30 seconds (the average time taken for easy separation of the gel cap in a pre-study sample of 20 gels caps) before the endoscope was withdrawn and the feeding tube advanced into the stomach. There were no adverse events in either group.

The outcome measures recorded by medical staff were ease of procedure (graded 0-100 on a visual analogue scale (VAS) where 0 is very easy and 100 is very difficult), perceived patient discomfort (VAS 0-100 where 0 is no discomfort and 100 is very uncomfortable), length of procedure (seconds) and number of assistants required to facilitate insertion. All procedures were carried out by one of two clinicians experienced in using both techniques.

Following insertion, patients were asked to complete a further VAS to grade their pain score (VAS 0-100; 0 is no pain and 100 is worst pain ever) and discomfort (VAS 0-100; 0 is no discomfort and 100 is most uncomfortable thing ever). The clinician performing the follow-up patient questionnaire was blind to the technique used in order to reduce bias whilst collecting results. The data was normally distributed having passed normality testing (alpha = 0.05).

## Audit outcome

Thirty-five patients (27 males, 8 females) were included in this 10-month comparative audit. All patients were eligible following failure of traditional nasogastric tube insertion techniques. Following inclusion, patients were randomised into one of two groups (A or B).

### Group A

Seventeen patients underwent endoscopic insertion without the gel cap. Of these, the procedure initially failed on 5 occasions. One patient was withdrawn as he could not tolerate any procedure and required insertion under general anaesthesia. On four occasions, passage of the feeding tube through the nose was not possible. Three of these patients had large nasopharyngeal tumours and one had previously sustained a nasal fracture and had deranged intranasal anatomy. All four patients subsequently had tubes passed successfully using the gel cap to aid manoeuvrability through the nose.

### Group B

Eighteen patients were allocated to undergo insertion using the gel cap. The procedure failed on five occasions. In three patients, the gel cap became dislodged in the nasopharynx and the procedure was completed under direct vision via the flexible nasendoscope without requiring withdrawal of the endoscope or feeding tube. In two patients the endoscope and feeding tube in combination was too large to pass through the nose due to deranged anatomy from previous trauma. Both patients had NG tube insertion without using the gel cap and without complications.

Table 1 demonstrates baseline variables for each group. Table 2 outlines the indications for insertion of nasogastric feeding tube for each group.

**Table 1:** Patient Demographics

Demographics	Without Cap (Group A)	With Cap (Group B)
Randomised	17	18
Sex (M : F)	13 : 4	14 : 4
Age (mean, range)/years	72 (32-90)	69.5 (44-88)

**Table 2:** Indication for Insertion of NG Feeding Tube

Indication	Without Cap (Group A)	With Cap (Group B)
Laryngeal cancer. Aspiration pre op	5	5
Laryngeal cancer. Aspiration post op	5	5
Tumour of tongue base	1	1
Cachectic pre treatment	1	2
Dysphagia post radiotherapy	1	-
Nasopharyngeal cancer	3	1
Tongue swelling post photodynamic therapy for tongue tumour	-	2
Other	1	2

The clinician and patient rating for discomfort was similar but there was no significant association between perceived discomfort ratings and actual pain scores, although there was a trend for both patients (p = 0.0110 Mann Whitney test) and clinicians (p = 0.0234 Mann Whitney test) to predict how much pain patients would report (Table 3). There was a significant correlation between time taken and perceived ease of the procedure (no cap R^2 ^0.4366, cap R^2 ^0.6540) as well as number of attempts with both time (caps R^2 ^0.7997, no caps R^2 ^0.4578) and perceived ease (cap R^2 ^0.4628) but not for procedures with the use of the cap coupling technique (no cap R^2 ^0.1173). There was no significant correlation between reported pain scores and time taken in either group. The perceived ease of procedures and both the clinician's and patients perception of perceived discomfort of the procedure were correlated with reported pain (Person's correlation coefficient p < 0.05), (Table 3). Although there was no significant difference in the time taken for the procedure in either group, group B (gel cap coupling) had an inbuilt 30-second wait before consideration was given to withdrawing the scope after successful placement. Otherwise no significant difference between the two techniques was identified. It may have been redundant to ask both discomfort and pain scores but we felt this covered the spread of patient perception since pain is by its very nature subjective.

**Table 3:** Primary Outcome Measures

Outcome measure	Group A (n = 17)	Group B (n = 18)	Mann Whitney p-Value Z scores
Ease of Procedure (0-100)	Median = 58	Median = 41	p 0.2985
	Mean = 59.94	Mean = 48.72	z 1.043280466
	Std = 31.58	Std = 32.03	
			
Patient discomfort score (0-100)	Median = 46	Median = 32.5	p 0.3728
	Mean = 48.47	Mean = 42.11	z 0.653648012
	Std = 28.77	Std = 28.77	
			
Length of procedure (seconds)	Median = 225	Median = 145	p 0.1866
	Mean = 244.1	Mean = 181.7	z 1.268717
	Std = 163.1	Std = 124	
			
Patent pain score (0-100)	Median = 20	Median = 23.5	p 0.8559
	Mean = 32.06	Mean = 32.5	z 0.039598998
	Std = 33.9	Std = 31.71	
			
Dr perceived discomfort (0-100)	Median = 50	Median = 31	p 0.5524
	Mean = 50	Mean = 43.06	z 0.623254274
	Std = 34.38	Std = 31.31	
			
Assistance required	9/17	3/18	z 0.0625
			
Success only after converting to alternative technique	5/17	4/18	p 0.5718
			
No success either technique	1	0	-

## Discussion

The practice of caution before fully instituting research findings is a common surgical trait. This is to ensure patients are protected from the vagaries investigative results, since these may not translate easily into useful applications for patients, their pathology and available resources. We illustrate this point with our attempt to utilise a simple method of inserting NG tubes still not commonly employed despite its description some years ago. Reducing risk from this relatively simple intervention is a desirable outcome [7].

In this case we found the methodology to be of no greater benefit to our patients when compared to our alternative current practice for failed blind NG tube insertion. The research had already been performed by Sjouri et al., was identified as possibly better practice (an audit criteria). However rather than throwing the 'baby out with the bathwater' we carried out a comparative audit against our current practice and finding it to be indeed an alternative for our patient population we have adopted its usage for certain circumstances. Although intuitively more attractive we could find no evidence that it was better.

One may question whether this is true audit in its purest form or just local adaptation of research findings rather than a blind adoption of technique. This audit was small but our randomisation was rigorous. Despite this, our study could be prone to confounding effects as well as type 1 and 2 errors. A comparative audit on all patients requiring a nasogastric tube rather than a selected subset of failures of 'blind' placement was contemplated, however since most tubes can be placed 'blindly' with no problems there were several reasons for not pursuing this course of investigation. In difficult cases perhaps a multicentre trail would prove more beneficial if clear outcomes and patient benefit and cost reductions could be found.

We retain this methodology in our armamentarium for difficult circumstances but have continued with our standard practice for most patients needing nasogastric tube placement.

## Competing interests

The authors declare that they have no competing interests.

## Authors' contributions

TU, PS, MC, JM, HT, and WKJ: designed the study, carried out data collection, literature research, manuscript preparation and manuscript review. All authors read and approved the final manuscript.
